# Long-term safety of paclitaxel drug-coated balloon-only angioplasty for de novo coronary artery disease: the SPARTAN DCB study

**DOI:** 10.1007/s00392-020-01734-6

**Published:** 2020-09-02

**Authors:** Ioannis Merinopoulos, Tharusha Gunawardena, Upul Wickramarachchi, Paul Richardson, Clint Maart, Sulfi Sreekumar, Chris Sawh, Trevor Wistow, Toomas Sarev, Alisdair Ryding, Tim Gilbert, Aris Perperoglou, Vassilios S. Vassiliou, Simon C. Eccleshall

**Affiliations:** 1 Department of Cardiology, Norfolk and Norwich University Hospital, Norwich, UK; 2 Norwich Medical School, University of East Anglia, Norwich, UK; 3 School of Mathematics, Statistics and Physics, Newcastle University, Newcastle Upon, UK; 4 Royal Brompton Hospital, London, UK; 5 Norwich Medical School, University of East Anglia, 2.06 Bob Champion Research & Education Building, Norwich, NR4 7TJ UK

**Keywords:** Stable angina, Mortality, Drug-coated balloon

## Abstract

**Objectives:**

We aimed to investigate long-term survival of paclitaxel DCB for percutaneous coronary intervention (PCI).

**Background:**

Safety concerns have been raised over the use of paclitaxel devices for peripheral artery disease recently, following a meta-analysis suggesting increased late mortality. With regard to drug-coated balloon (DCB) angioplasty for coronary artery intervention however, there is limited data to date regarding possible late mortality relating to paclitaxel.

**Methods:**

We compared all-cause mortality of patients treated with paclitaxel DCB to those with non-paclitaxel second-generation drug-eluting stents (DES) for stable, de novo coronary artery disease from 1st January 2011 till 31st December 2018. To have homogenous groups allowing data on safety to be interpreted accurately, we excluded patients with previous PCI and patients treated with a combination of both DCB and DES in subsequent PCIs. Data were analysed with Kaplan–Meier curves and Cox regression statistical models.

**Results:**

We present 1517 patients; 429 treated with paclitaxel DCB and 1088 treated with DES. On univariate analysis, age, hypercholesterolaemia, hypertension, peripheral vascular disease, prior myocardial infarction, heart failure, smoking, atrial fibrillation, decreasing estimated glomerular filtration rate (eGFR) [and renal failure (eGFR < 45)] were associated with worse survival. DCB intervention showed a non-significant trend towards better prognosis compared to DES (*p* = 0.08). On multivariable analysis age, decreasing eGFR and smoking associated with worse prognosis.

**Conclusion:**

We found no evidence of late mortality associated with DCB angioplasty compared with non-paclitaxel second-generation DES in up to 5 years follow-up. DCB is a safe option for the treatment of de novo coronary artery disease.

**Electronic supplementary material:**

The online version of this article (10.1007/s00392-020-01734-6) contains supplementary material, which is available to authorized users.

## Introduction

Drug-coated balloons (DCB) are an emerging PCI technology negating the need for stent implantation [1–3]. Thus far, it has an established role in the treatment of in-stent restenosis [4] with a growing number of studies showing excellent results in de novo coronary artery disease [5–9]. The great majority of DCB used are coated with paclitaxel, but encouraging results have emerged over the last year for the use of sirolimus-coated balloons in coronary artery disease [10, 11]. However, a recent systematic review and meta-analysis of summary-level data raised concerns about the use of paclitaxel-containing devices for peripheral arterial disease, suggesting a signal of increased late mortality associated to the paclitaxel dose–time product [12]. Subsequent studies however, with individualised-data analysis of patients treated with paclitaxel DCB for peripheral arterial disease demonstrated no difference in all-cause mortality between DCBs and uncoated percutaneous transluminal angioplasty [13, 14]. Despite not universal, this concern was sufficient for the FDA to initiate an investigation for the use of paclitaxel-containing devices for peripheral arterial disease [15]. Currently, there are no data on long-term results of paclitaxel DCB used to treat de novo coronary artery disease. Moreover, the dose of paclitaxel in coronary DCBs (0.3-0.6 mg) is at least an order of magnitude lower compared with paclitaxel-eluting devices (8.5 mg for IN.PACT 6 × 120 mm balloon for example) for peripheral artery disease [12, 16] indicating that any results from peripheral DCB cannot be extrapolated to coronary DCB. In our study, we aimed to explore whether there is a signal of increased late mortality in patients treated with paclitaxel DCB for de novo coronary artery disease in up to 5-year follow-up.

## Methods

**Fig. 1:**
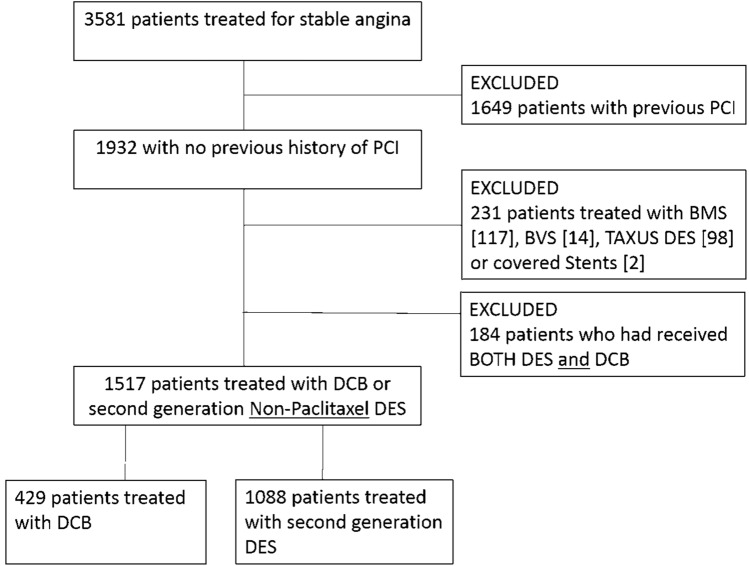
Study consort diagram. Consort diagram indicating how the final population included in the study was identified

The long-term Safety of PAclitaxel dRug coaTed balloon only ANgioplaSty for de novo coronary artery disease (SPARTAN DCB) study was an investigator-initiated, single-centre, cohort study. In our institution, patients treated with PCI are collated prospectively in a dedicated database. Following approval from the Northwest Haydock research ethics committee and institutional approval from the Norfolk & Norwich University Hospital, we retrospectively surveyed our clinical database to identify all patients treated with either paclitaxel DCB or 2nd-generation non-paclitaxel drug-eluting stents for stable, de novo coronary artery disease between 1st January 2011 and 31st December 2018. Due to the retrospective nature of our study, the confidentiality advisory group waived the need for patient consent. In order to investigate the true potential effect of paclitaxel and to achieve as homogenous a group as possible from our real-world data, we excluded patients being treated for ST elevation myocardial infarction (STEMI) or non-ST elevation myocardial infarction (NSTEMI). We also excluded patients with prior PCI to ensure homogeneity of our cohort. Similarly, we excluded patients who had repeat PCIs following their index procedure if the PCI strategy was different to the index procedure: i.e. patients treated with DES initially and then later treated with DCB or vice versa were excluded as shown in the consort diagram (Fig. 1); however, if the patients received a DES or DCB on all occasions they were not excluded. Clinical and angiographic data were obtained from our prospectively collated database and supplemented with data from electronic records where required. The vessel diameter was taken as the largest pre/post-dilatation balloon, DCB or DES used while lesion length was based on the DCB or DES length.

The primary endpoint was all-cause mortality. Survival data were obtained through the UK Health and Social Care Information Service, an independent national body where all deaths in the UK are recorded by law. Mortality data were obtained 6 months following the last study patient to ensure a minimum of 6-month follow-up for every patient.

Statistical analysis was undertaken in program R (version 3.6.0) by an independent professional statistician. Nominal variables were reported as counts and percentages and compared by the Chi-square and Student’s *t* test as appropriate. Kaplan–Meier estimator curves were used to plot survival. For the main analysis, all-cause mortality was limited to 5 years post-index procedure (if a patient died beyond 5 years follow-up, they were considered alive for the purposes of this analysis) in order to minimise the difference in follow-up between DES and DCB group. Kaplan–Meier survival curves were also plotted for those patients alive at 2 years in order to specifically investigate a late paclitaxel effect. Comparisons were performed by the log-rank test. Univariate and multivariate Cox regression analyses were performed to identify predictors of mortality.

## Results

A total of 429 consecutive patients treated with paclitaxel DCB and 1088 consecutive patients treated with non-paclitaxel 2nd-generation DES were identified (Fig. 1). Some 94% of patients in the DCB group were treated with iobromide paclitaxel DCB (67.4% SeQuent Please NEO and 26.6% SeQuent Please), 5% with urea paclitaxel DCB (Falcon) and 1% with other paclitaxel DCB. Some 33.5% of patients were treated with Promus Premier and 26.8% with Promus Element DES, 13% with Synergy DES, 7.6% with Xience Prime, 5.7% with Xience Pro, 6.5% with Onyx DES, 2.9% with Ultimaster DES, 1.8% with Combo dual therapy DES and 2.2% with other second-generation DES. The average age was 66.9 ± 10.2 and 66.8 ± 10 years old for the DCB and DES group, respectively. Male patients accounted for 76.2% of the DCB group and 76.6% of the DES. Table 1 demonstrates that the two groups were well balanced for the great majority of baseline patient characteristics. The DES group had a significantly higher incidence of patients with chronic obstructive pulmonary disease and smoking history while the DCB group had a significantly higher incidence of patients with atrial fibrillation. Significantly more patients were on dual antiplatelet therapy (DAPT) in the DES group and as expected the mean duration of DAPT was significantly longer in the DES group.

**Table 1:** Baseline patient characteristics of study groups

	Paclitaxel DCB (*n* = 429)	Non-paclitaxel 2nd-generation DES (*n* = 1088)	*p*-value
Age	66.9 ± 10.2	66.8 ± 10	0.79
Male	327 (76.2)	834 (76.6)	0.86
Hypercholesterolaemia	161 (37.5)	456 (41.9)	0.12
Hypertension	236 (55.0)	639 (58.7)	0.19
Peripheral vascular disease	17 (3.9)	48 (4.4)	0.69
Cerebrovascular event	30 (6.9)	54 (4.9)	0.15
Myocardial infarction	52 (12.1)	167 (15.3)	0.11
Coronary artery bypass	35 (8.1)	82 (7.5)	0.68
Heart failure	14 (3.2)	40 (3.6)	0.69
Family history of IHD	133 (31.0)	324 (29.7)	0.64
COPD	14 (3.2)	66 (6.0)	0.02*
Diabetes	98 (22.8)	229 (21.0)	0.44
Smoking (current/previous)	247 (57.5)	696 (63.9)	0.02*
Atrial fibrillation	37 (8.6)	40 (3.6)	< 0.01*
eGFR	78.8 ± 20.1	78.5 ± 21.1	0.81
DAPT	397 (92.5)	1050 (96.5)	< 0.01*
Mean DAPT duration	73.5 ± 104.7	355.6 ± 60.5	< 0.01*

Baseline patient characteristics of patients treated with DCB or DES. Data are n (%) and *denotes significant result

*COPD* chronic obstructive pulmonary disease, *IHD* ischaemic heart disease, *eGFR* estimated glomerular filtration rate, *DAPT* dual antiplatelet therapy

Table 2 shows the characteristics of the target vessels treated with DCB or DES. The groups were well balanced in terms of prognostically significant lesions targeted with no difference in left main coronary artery, left anterior descending artery or multi-vessel PCI.

**Table 2:** Target vessels of study groups

	Paclitaxel DCB (*n* = 429)	Non-paclitaxel 2nd-generation DES (*n* = 1088)	*p* value
LMS	10 (2.3)	34 (3.1)	0.41
LAD	229 (53.4)	545 (50.1)	0.25
Cx	76 (17.7)	135 (12.4)	< 0.01*
RCA	77 (17.9)	250 (22.9)	0.03*
Graft	4 (0.9)	27 (2.4)	0.06
Multi-vessel PCI	33 (7.7)	97 (8.9)	0.44
Mean vessel diameter, mm	3.06 ± 0.56	3.39 ± 0.59	< 0.01*
Mean lesion length, mm	26.05 ± 11.95	30.03 ± 16.52	< 0.01*
Large vessels (diameter ≥ 3 mm)	320 (74.6)	925 (85)	< 0.01*

Target vessels treated with DCB or DES

*LMS* left main stem, *LAD* left anterior descending artery, *Cx* circumflex, *RCA* right coronary artery, *PCI* percutaneous coronary intervention. Data are n (%) and *denotes significant result

The patients were followed up for an average of 31.6 ± 16.3 months (interquartile range 16.8–45.3 months) in the DCB group and 44.4 ± 18.4 months (interquartile range 27.1–60 months) in the DES group. We obtained mortality data for 1515 patients. It was not possible to obtain mortality status of two patients (one in each group) who were censored at the time of last known alive.

**Fig. 2:**
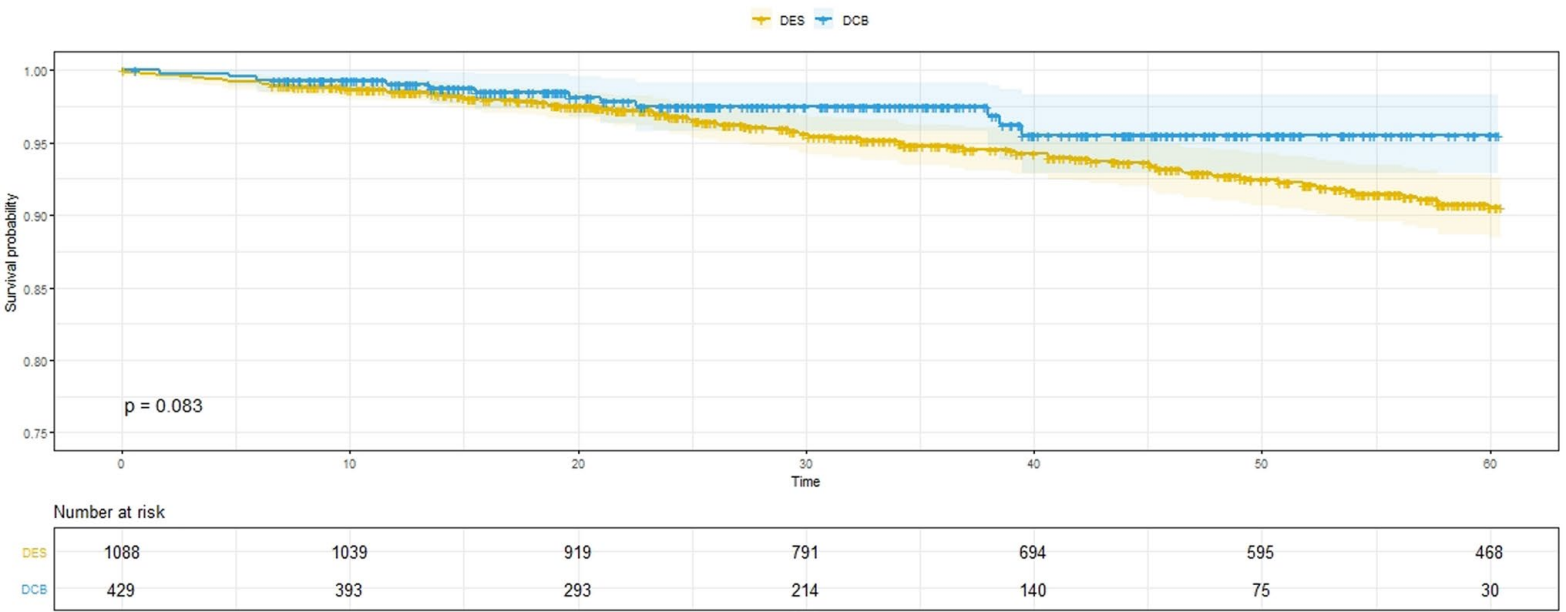
Kaplan–Meier estimator plot. Kaplan–Meier estimator plot of all-cause mortality for paclitaxel DCB versus non-paclitaxel 2nd-generation DES with numbers at risk are shown below the graph. *DCB* drug-coated balloon, *DES* drug-eluting stent

**Fig. 3:**
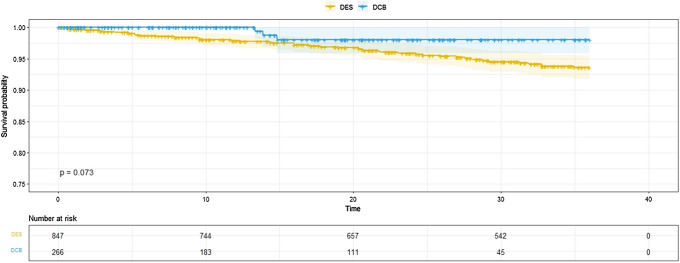
Kaplan–Meier estimator plot of patients alive at 2 years. Kaplan–Meier estimator plot of patients alive at 2 years showing no significant difference between DES and DCB, with numbers at risk shown below the graph. *DCB* drug-coated balloon, *DES* drug-eluting stent

There was no evidence of increased late mortality associated with paclitaxel DCB for de novo coronary artery disease compared with non-paclitaxel 2nd-generation DES (Fig. 2). Interestingly, the Kaplan–Meier curves separate early and then continue to diverge; supporting that DCB-only angioplasty is a safe procedure. Analysis following propensity score matching supported these results (Supplementary Fig. 1). The supplementary Table I demonstrated the 30-day, 6, 12, 24, and 36-month mortality in the DCB and DES groups. After 36 months of follow-up, 9 patients died in the DCB group vs 50 patients in the DES group. We specifically investigated a possible late mortality effect by analysing separately those patients who were alive 2 years following the index PCI and there was no evidence of increased late mortality with paclitaxel DCB (Fig. 3).

Univariate Cox regression analysis identified the following adverse prognostic factors: age, hypertension, peripheral vascular disease, previous myocardial infarction, heart failure, smoking, atrial fibrillation and decreasing estimated glomerular filtration rate (eGFR) [and renal failure defined as estimated glomerular filtration rate (eGFR) < 45] (Table 3). Hypercholesterolaemia and family history of ischaemic heart disease were associated with better prognosis on univariate analysis (Table 3). None of the angiographic characteristics were associated with worse outcome. On multivariate Cox regression analysis only age, decreasing eGFR [and renal failure defined as eGFR < 45—not presented in Table 4] and smoking history remained significant poor prognostic factors (Table 4).

**Table 3:** Univariate Cox regression analysis

Variable	*p* value	HR [95% CI]
DCB	0.08	0.765 [0.56, 1.04]
Female	0.08	1.496 [0.95, 2.35]
Age	< 0.01*	1.115 [1.08, 1.14]
Hypercholesterolaemia	0.01	0.566 [0.36, 0.89]
Hypertension	0.01*	1.808 [1.14, 2.85]
Peripheral vascular disease	< 0.01*	2.674 [1.34, 5.32]
Cerebrovascular disease	0.81	0.882 [0.32, 2.40]
Myocardial infarction	0.03*	1.716 [1.05, 2.80]
Heart failure	< 0.01*	3.439 [1.66, 7.12]
Family history of IHD	< 0.01	0.434 [0.24, 0.75]
Diabetes	0.23	1.345 [0.83, 2.17]
COPD	0.40	1.426 [0.62, 3.26]
Smoking	< 0.01*	1.965 [1.20, 3.20]
BMI	0.26	0.975 [0.93, 1.02]
Atrial fibrillation	< 0.01*	3.151 [1.57, 6.31]
eGFR	< 0.01*	0.969 [0.959, 0.979]
Renal failure (eGFR < 45)	< 0.01*	4.997 [2.94, 8.49]
CABG	0.27	1.453 [0.75,2.80]
DAPT duration	0.65	1.000 [0.998, 1.001]
LMS	0.11	2.100 [0.85, 5.17]
LAD	0.12	0.714 [0.46, 1.08]
Cx	0.07	1.621 [0.96, 2.71]
RCA	0.78	0.931 [0.56, 1.54]
Graft	0.49	1.495 [0.47, 4.72]
Multi-vessel PCI	0.72	0.868 [0.40, 1.87]
Vessel diameter	0.59	1.101 [0.77, 1.55]
Lesion length	0.41	0.995 [0.984, 1.007]

Results of univariate Cox regression analysis

*IHD* ischaemic heart disease, *COPD* chronic obstructive pulmonary disease, *BMI* body mass index, *CABG* coronary artery bypass graft, *DAPT* dual antiplatelet therapy, *LMS* left main stem, *LAD* left anterior descending, *Cx* circumflex, *RCA* right coronary artery, *PCI* percutaneous coronary intervention

*Denotes adverse prognostic factor

**Table 4:** Multivariate Cox regression analysis

Variable	*p* value	HR [95% CI]
Age	< 0.01*	1.087 [1.06, 1.12]
Heart failure	0.19	1.653 [0.77, 3.55]
eGFR	0.01*	0.985 [0.974, 0.997]
Family history of IHD	0.56	0.843 [0.47, 1.51]
Hypertension	0.11	1.456 [0.91, 2.32]
Hypercholesterolaemia	0.11	0.683 [0.43, 1.09]
Peripheral vascular disease	0.45	1.340 [0.63, 2.84]
Smoking	0.01*	1.925 [1.17, 3.16]
Myocardial infarction	0.16	1.439 [0.87, 2.39]
CABG	0.68	0.865 [0.44, 1.71]
Atrial fibrillation	0.32	1.450 [0.70, 3.00]

Results of multivariate Cox regression analysis. IHD= ischaemic heart disease, CABG: coronary artery bypass graft. *Denotes adverse prognostic factor

## Discussion

Drug-coated balloon-only angioplasty is recommended by evidence-based guidelines for the treatment of in-stent restenosis while there is also evidence to support their use in small-vessel disease and patients with high bleeding risk [5, 17, 18]. Following a recent meta-analysis though, concerns have been raised regarding the safety of paclitaxel devices for peripheral artery disease [12]. In SPARTAN DCB study, paclitaxel DCB was not associated with increased late mortality, up to 5 years of follow-up. Instead, there was a trend for better survival when compared with second-generation DES.

Our results are consistent with two recent meta-analyses. The recent DAEDALUS study in patients treated with DCB or DES for in-stent re-stenosis showed that there was no significant difference in late mortality associated with DCB. This conclusion is limited however, by the fact that follow-up was limited to 3 years and thus might have missed a true late effect [19]. In addition, it is difficult to draw definitive conclusions from that study for late mortality relating to paclitaxel, as this was a subgroup analysis and the patient groups were heterogeneous given the previous stent implantations including bare metal stents and paclitaxel DES. A most recent meta-analysis specifically investigating the mortality of paclitaxel DCB for coronary intervention did not show increased mortality with DCB [20]. However, this meta-analysis included significantly heterogeneous studies comparing paclitaxel DCB with control treatments such as plain old balloon angioplasty, bare metal stents, paclitaxel and non-paclitaxel drug-eluting stent mostly in the setting of in-stent restenosis.

In the SPARTAN DCB study, we included large numbers of patients treated for de novo coronary artery disease and ensured homogeneity of the groups by excluding patients with previous PCI or patients who received both DCB and DES either at their index or subsequent PCIs. As such, our groups of DCB and DES were well-matched for patient characteristics and angiographic findings. We have demonstrated that there is no evidence of increased late mortality associated with paclitaxel DCB compared to non-paclitaxel second-generation DES for de novo coronary artery disease up to 5 years of follow-up. In fact, there was actually a trend towards better survival with DCB, a finding consistent with the most recent meta-analysis [20]. Furthermore, we specifically investigated a late paclitaxel effect by analysing only patients who were alive at 2 years, with no evidence of increased late mortality associated with paclitaxel DCB either.

Following a meta-analysis raising concerns about a possible long-term mortality signal due to paclitaxel-eluting devices for peripheral vascular disease [12], an intense debate about the conclusion and various limitations of that study has been triggered in the literature [11, 21–23]. Whilst subsequent studies have failed to confirm these initial concerns, the FDA has nonetheless initiated an ongoing investigation for this matter [15]. Despite the similarities in peripheral and coronary DCB, there are also major differences. For example, the dose of paclitaxel in DCBs for coronary artery disease is about an order of magnitude lower compared to the dose of paclitaxel in paclitaxel-coated devices for peripheral artery disease [16] making it therefore unclear whether, even if the results of the DCB for peripheral vascular disease were adverse, how this would translate to the coronary DCB PCI. Furthermore, the underlying mechanism leading to a possible increased late-mortality signal with DCB for peripheral artery disease remains to be defined. Nevertheless, given that the outcomes that were notably concerning included cardiovascular mortality, it is crucial to study the results of paclitaxel DCB for coronary artery disease carefully and provide assurance of safety.

## Limitations

The retrospective, non-randomised nature of our work from a single centre can introduce referral bias. However, our institution is a large tertiary referral centre providing cardiac intervention to a population in excess of one million, with the highest implantation of DCBs for coronary artery disease in the UK [24], and we included all consecutive patients fulfilling the criteria. However, our results might not be generalisable to smaller institutions with less experience with DCB-only angioplasty. Even though our study is retrospective and non-randomised, our clinical database was completed prospectively and the two groups were well balanced in terms of patient and angiographic characteristics. The DES group had significantly longer follow-up, but this was mitigated by limiting the analysis to 5 years post-index procedure (if a patient died beyond 5 years follow-up, they were considered alive for the purposes of this study).

## Conclusion

In conclusion, this is the first study to specifically report on the long-term 5-year follow-up of patients undergoing elective DCB PCI for stable, de novo, coronary artery disease and compared with second-generation non-paclitaxel stents. Our study shows that there is no evidence of increased late mortality associated with paclitaxel DCB for stable, de novo coronary artery disease and therefore, DCB could be considered in this population.

## Electronic supplementary material

Below is the link to the electronic supplementary material.

Supplementary material 1 (TIFF 126 kb)

Supplementary material 2 (DOCX 14 kb)

## Data Availability

Data can be available following appropriate request to the authors.
