# Supplementary figures and images for: Long-term safety of paclitaxel drug-coated balloon-only angioplasty for de novo coronary artery disease: the SPARTAN DCB study
Source: Clin Res Cardiol. 2020 Sep 2;110(2):220–7. doi: 10.1007/s00392-020-01734-6 (PMC7862512; doi:10.1007/s00392-020-01734-6)

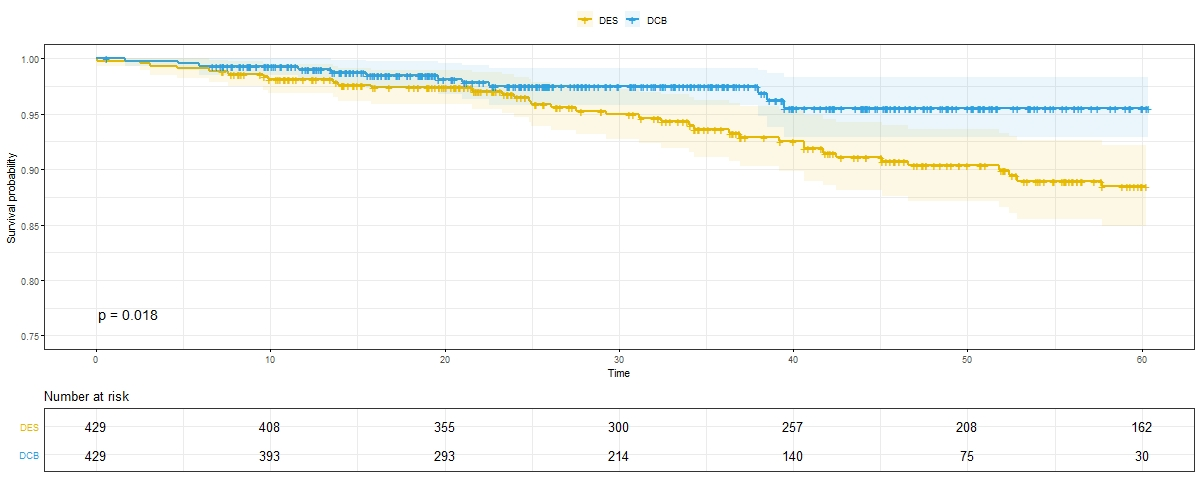

Supplement: Supplementary file 1 — Supplementary material 1 (TIFF 126 kb) [file 392_2020_1734_MOESM1_ESM.tif]
