# Supplementary material for: Long-term safety of paclitaxel drug-coated balloon-only angioplasty for de novo coronary artery disease: the SPARTAN DCB study
Source: Clin Res Cardiol. 2020 Sep 2;110(2):220–7. doi: 10.1007/s00392-020-01734-6 (PMC7862512; doi:10.1007/s00392-020-01734-6)
Supplement: Supplementary file 2 — Supplementary material 2 (DOCX 14 kb) [file 392_2020_1734_MOESM2_ESM.docx]

**Supplementary table I. Title: Mortality rate of study groups**

| **Mortality (time)** | **DCB mortality** | **DCB number at risk** | **DCB mortality / DCB number at risk (%)** | **DCB mortality / (DCB number at risk + DCB mortality) (%)** | **DES mortality** | **DES number at risk** | **DES mortality / DES number at risk (%)** | **DES mortality / (DES number at risk + DES mortality) (%)** |
| --- | --- | --- | --- | --- | --- | --- | --- | --- |
| **30 day** | 0 | 428 | 0% | 0% | 3 | 1084 | 0.3% | 0.3% |
| **6 months** | 3 | 425 | 0.7% | 0.7% | 10 | 1077 | 0.9% | 0.9% |
| **12 months** | 4 | 371 | 1.1% | 1.1% | 16 | 1016 | 1.6% | 1.6% |
| **24 months** | 9 | 266 | 3.4% | 3.3% | 33 | 847 | 3.9% | 3.8% |
| **36 months** | 9 | 165 | 5.6% | 5.2% | 50 | 723 | 6.9% | 6.5% |

**Supplementary table I: 30-day, 6, 12, 24 and 36 month mortality in DCB and DES groups.**
